# Making legal sense: on jurors’ discovery of objectivity in Argentina’s experience of lay participation in criminal trials

**DOI:** 10.3389/fsoc.2025.1435354

**Published:** 2025-07-08

**Authors:** Santiago Abel Amietta

**Affiliations:** School of Social Sciences, Keele University, Newcastle-under-Lyme, United Kingdom

**Keywords:** lay participation, legal consciousness, jurors, objectivity, justice, emotions, Argentina

## Abstract

This article stems from a broader research programme on the recent incorporation of lay decision-makers into the historically professional-only criminal justice systems in Argentina. It draws on ethnographic data from courthouse observations and in-depth interviews with ordinary citizens who served as lay jurors in the mixed tribunal of the Province of Córdoba, the first one in the country to introduce lay participation. The article deploys the conceptual framework of relational legal consciousness to examine jurors’ perceptions of their own role and experiences within the courthouse, vis-à-vis legal professionals and their deployment of legal knowledge. It argues that jurors’ stories of the use of the law, its language and formalities complicate their perception, in conventional and scholarly wisdom, as bearers of emotions and common sense—a realm opposed to the one imagined and reserved for legal professionals, the sphere of uncontaminated application of legal rules and principles. The article contributes in this way to broader debates on the place and impact of lay decision-makers on state judicial adjudication and on the role of emotions and extra-legal reasoning therein.

## 1 Introduction

Criminal jury trials have been incorporated in every version of Argentina’s National Constitution since its transition to formal postcolonial independence in the first half of the 19th century. Until the 1980s, however, the implementation of lay participation in criminal trials was rarely addressed in spheres of authorized legal discourse (Hendler, 2008; Bergoglio, 2010; Bergoglio et al., 2019). With the return of democratic rule following the last civic-military dictatorship (1976–1983), a process of legal and judicial reforms ensued. These reforms included profound modifications to the federal and provincial criminal procedure systems—decisively shifting from an inquisitorial to an adversarial style, and from written to oral proceedings. While lay participation was not central to this wave of reforms, discussions about its introduction gained visibility. In 1991, the Province of Córdoba passed the first system (a mixed tribunal of German inspiration, with two laypeople and three professional judges) and introduced a new one in 2004, with expanded lay participation. In its current form, eight lay people^1^ are summoned to take part in the decision of a single case (the most serious murders and cases of public officials’ corruption), sitting with three judges and deciding the verdict by the majority.

This article draws on interviews with individuals who participated as jurors in the first decade of Córdoba’s majority-lay mixed tribunal to examine their perceptions of their own role and the ways ‘objectivity’ is deployed in the judicial setting. It looks at stories of their use of legal and extra-legal knowledge to define their own sense of justice. Empirical ‘law and emotions’ studies that focus on adjudicators tend to look at the “subtle and repeatedly negotiated relations between legal thinking and ideas of detachment and disinterest in judicial decision-making contexts, on the one hand, and emotions and their expressions or recognition, on the other hand” (Vaisman and Barrera, 2020: 813, see also Roach Anleu and Mack, 2019). This tendency has special implications in the case of lay jurors who are professionally and ethically less constrained than judges to leave emotions aside. They are ambiguously situated both in the spaces and routines of courthouses and at the intersection of the taken-for-granted divide between legal reasoning and common sense (Amietta, 2019, 2021). Bringing relational, affective, and emotional factors on board complicates this ambiguity further, as put by Hastie:

There is an apparent contradiction between the conception of the ideal juror as a logical reasoning machine and as a source of community attitudes, sentiments, and moral precepts. Robert Solomon noted this discrepancy when he commented that “[the idea that justice requires emotional detachment, a kind of purity suited ultimately to angels, ideal observers, and the original founders of society, has blinded us to the fact that justice arises from and requires such feelings as resentment]” (Hastie, 2001, pp. 991–992).

Given this uniquely ambiguous status of lay decision-makers, it is not surprising that questions of the weight of emotions in their decision-making have been commonplace in lay participation research since long before the emergence ‘law and emotions’ as a scholarly field (Maroney, 2016). Ever since the inception of socio-legal research on juries through Kalven and Zeisel’s (1996) *The American Jury* (1966), the question of emotions has remained explicitly or implicitly central to a field that has primarily aimed to empirically establish the true nature of lay decision-makers’ contributions to state justice systems (see, for example, Diamond and Rose, 2005; Eisenberg et al., 2005; Ellison and Munro, 2010; Gastil and Weiser, 2006).

The aim of this article differs from this line of analysis for methodological and epistemological reasons. Methodological reasons are straightforward. I refrain from trying to identify “paths of emotional influences” or “measuring emotional effects” on jurors’ decisions (Feigenson, 2016), in part because I did not have access to the deliberations of mixed tribunals. While in Córdoba’s system, the votes of both professional and lay members are made public with a motivation written by the presiding judge—a document that makes for an original and interesting legal artifact, they provide only a very limited glimpse of the process. I resort, instead, to interviews with individuals who are among the first few thousand to have taken part in criminal adjudication in a historically professional-only justice system to explore a different question: how do jurors discover, relate to, understand, and deploy (or choose not to deploy) law’s own brand of objective decision-making? I investigate how they act in the face of it, and what this means for their experience of participation.

My focus on the relationship of lay adjudicators with legal institutions, their uses of law and legal knowledge and the affective dimensions of the experience in and beyond courthouses is underpinned by literature on legal consciousness. The relationship of common citizens with law and legal institutions has been, for at least three decades, especially in the USA, the object of much socio-legal scholarly attention. Interested in decentering capital-L ‘Law’ in law and society scholarship, studies of legal consciousness emerged in the 1980s searching for common people’s deployment of legal practices and meanings outside and beyond the workings of legal institutions, within myriad ‘everyday life’ settings (Greenhouse et al., 1994; Silbey, 2005; Williams, 1993). While much has been argued about the definition, actual ontological substance, and empirical and critical possibilities of the concept (Silbey, 2005), there seems to be some level of agreement that legal consciousness studies look at these phenomena from a critical point of view, but one situated in the middle ground existing between overwhelming arguments of structural, legal hegemony and the naïve individualism of Western legal formalist approaches. Consciousness is, in this subfield, “participation in this collective, social production of ideology and hegemony, an integral part of the production of the very same structures that are also experienced as external and constraining” (Silbey, 2005, pp. 333). Ordinary people, as they go about making legal claims, talking about law and rights, taking or choosing not to take legal action, reproducing or resisting those interventions in their own lives, do indeed make law.

In their book *The Common Place of Law* (1998), still the most influential theory-making effort in legal consciousness to date, Patricia Ewick and Susan Silbey criticized previous work looking at ordinary people’s relation to the legal system for treating legal consciousness, whether solely as attitude or as a mere epiphenomenon. Whereas the first conceptualization, reminiscent of liberal ideals, focused on individuals’ ideas and attitudes and ignored the structural constraints, the latter one, evocative of structural anthropology and Marxist structuralism, treated legal consciousness as a by-product of the operations of the social structure, leaving no space for any creative role of social actors who partake in the (re)production of social structures. Ewick and Silbey proposed instead an understanding of legal consciousness as a cultural analysis of legality that reciprocally integrates human action and structural constraint (1998: 34–43). Interpreting the way lines such as normativity, capacity, constraint, or time/space appeared in the narratives of their New Jersey interviewees, they theorized three underlying schemas into three forms of legal consciousness, or “commonplace stories of law” (Ewick and Silbey, 1998, pp. 223). Whereas in one schema, legality was displayed as a reified source of grandeur and principles (*Before the Law*), in another one, it appeared as a game to be strategically played in the pursuit of certain aims (*With the Law*), and finally as an oppressive force to be resisted against, largely through scarcely perceivable tactical maneuvers (*Against the Law*). This article follows and expands on these schemas, treating them not as a typology or set of rigid categories but as a springboard to expand this collective theory-making effort both conceptually and empirically.

With some notable exceptions (Fox, 2020), jurors have not been the usual protagonists of legal consciousness research. In their attempts to take the spotlight away from state law, authors in the field have largely identified ordinary citizens with the elusive notions of the ‘everyday’ and the ‘society’ as opposed to the sphere of official legal systems and institutions—‘The Law’ (Sarat and Kearns, 1993). Encompassing legal anthropology’s concern with dispute resolution, such identification is maintained when ordinary people choose to mobilize state legal systems’ apparatuses to solve their conflicts (Sarat and Kearns, 1993). As a result, when the loci have been sites of formal decision-making (Merry, 1990, Yngvesson, 1989) or other state agencies (Levi, 2009; Sarat, 1990), the focus has remained on the receiving end of the law–society divide: common citizens and their meaning-making processes upon encounters with such institutions as users of lower courts, mediation centers or state welfare agencies. While the introduction of lay participation is usually conceived by reformers and jury scholars as a way of bringing ‘society’ or ‘common sense’ into judicial decision-making, the identification of ordinary people with the ‘everyday’ and ‘society’ by legal consciousness studies seems to cease when it comes to jurors. The negative constitution of the everyday against the law (Valverde, 2003) situates jurors at the other extreme of the continuum, essentially delinked from *everyday* times, spaces and practices while they serve at the courthouse. When summoned as adjudicators, ordinary people become a constitutive part, if not an epitome, of the state legal systems that legal consciousness scholars define as their counterparts (Marcus, 1993; Sarat and Kearns, 1993; Silbey, 2005). The same reason that made jurors interested in *law and emotions* debates even before it developed as an autonomous socio-legal field accounts for the lack of scholarly attention to lay participation in judicial decision-making as a site for legal consciousness research. This article contributes to filling this gap.

Emotional and affective components of ordinary people’s relationship with the law have traditionally been part of the varied definitions of ‘legal consciousness’ in empirical socio-legal research (Harding, 2011; Marcus, 1993, Merry, 1990, Yngvesson, 1988). However, it is only recently that explicit bridges to the literature on law and emotions have been built by the wave of ‘relational legal consciousness’ studies that emphasize the affective factors involved in the constitution and performance of legal consciousness (Abrego, 2011, 2019; Wang, 2023; Wood, 2018; Young and Chimowitz, 2022). This perspective emphasizes the situated and fluid nature of legal consciousness along a number of lines, including people’s relationships and interactions with significant others (Liu, 2023, pp. 214). This article highlights this relational element in its discussion of the legal consciousness of jurors, and it puts the focus on jurors’ narrations of their relationship with legal professionals, in particular judges with whom they sat together in Córdoba’s mixed tribunals.

The discussion that follows is based on ethnographic research conducted in criminal trial courts in four locations of the Province of Córdoba—the Capital city and three smaller cities and towns. The research included an initial 7-month fieldwork conducted between October 2012 and April 2013, followed by two additional field visits in 2016 and 2023. Data collection consisted of a total of 62 interviews with officials from courts and other judicial offices, judges, prosecutors, lawyers, pro-jury activists, lawmakers, and individuals who served as jurors; close reading of documents (including case files and decisions, laws, draft laws, and transcripts of constitutional and legislative debates); and observation of courthouse routines and proceedings with and without lay participants. Interviews and archival research were also conducted in Buenos Aires and La Plata. This article relies mainly on semi-structured interviews I conducted with lay members of Córdoba’s mixed tribunals (*N* = 33) and my ethnographic field notes.^2^ These interviews navigated through a wide range of topics, including jurors’ experiences with legal institutions (before, during and after serving as decision**-**makers), their relationships with legal professionals at the courthouses, and their feelings during and after the proceedings. The analysis I present in this article focuses on jurors’ narratives of their understanding, uses and circulation of law, legal knowledge, and legal professionals. I argue that, as jurors discover the often intriguing and fluid workings of (legal) objectivity in court, most of them retreat to a position of awe and respect. Others, however, actively engage with this new understanding of the materiality and practice of objectivity, striving to embody and perform it in their roles and demanding it from legal professionals. This appropriation muddles the distinction between ‘common sense’ and ‘legal knowledge’ that is foundational to systems of lay participation and their socio-legal study. The findings are organized into two sections. In section 2, I discuss jurors’ understanding of their role, situation and status vis-à-vis the law and legal institutions—I will argue that jurors tend to situate themselves in generally passive positions in the face of the majesty and apparent complexity of the law and its processes. In section 3, I shift to stories of jurors consciously using, subverting, or going beyond legal mandates to pursue their agendas in ways that, I argue, problematize the law/common sense, lay/expert, and emotion/reason dichotomies. The concluding remarks discuss the article’s contributions and the implications of its findings.

## 2 Jurors, common sense, and an idea of justice

During the juror selection hearing for a murder case in the Capital City of Córdoba, a woman from an apparently lower socio-economic background requested to be exempted from serving. She was employed in two informal jobs and would have trouble keeping them if she missed a whole week. In addition, she had already served in one trial. Such requests are recurrent in the initial stages of the process and are usually adjudicated by court clerks informally depending on the number of jurors available and the reasons provided. “At the end of the day,” she told me aloud as the group waited in silence, “they already have everything done and bring us here just to help with the last little bit.” Amidst the indifference of other potential jurors, the middle-aged man working in car retail sitting next to me turned and quietly said, “just ignore her, she has not got a clue; good thing she is not staying.” The woman was, in fact, making a legally sound statement. Córdoba’s jurors are legally expected to serve only one time and her description of the jurors coming in to help with the last stage of the complex and often lengthy process of state punishment. However, in the view of another prospective juror, she was not in the know, which was a good reason for her to leave.

This ethnographic vignette provides some clues as to jurors’ relation to law, legal knowledge, and performances of objectivity, as well as the affective and relational dimensions of their experiences in the courthouse. Jurors’ stories convey a sense of ambiguity. On the one hand, law is and remains primarily a remote realm, detached from everyday life and burdened with hard-to-understand technicalities. Jurors’ ‘law talk’ overwhelmingly reproduces the conception of the law as an authoritative and predictable sphere separate from that of ordinary life. Put it in terms of Ewick and Silbey’s schema of legal consciousness, jurors I interviewed tend favor ‘Before the Law’ descriptions (Ewick and Silbey, 1998, pp. 47). Participation, as much as it implies commitment, knowledge and responsibility, is experienced by jurors as an invitation to witness (and bear witness to) the grandeur of ‘capital-L’ Law.

This distant working of the law emerges first, as embodied by judges and other legal professionals. Lay jurors in Córdoba’s mixed tribunals are accorded by the law, and for the duration of trials, they have equal status, with judges as members of the tribunal. They are granted the same protection and (symbolic, if not material) entitlements. For jurors, however, attributes such as knowledge of the law and experience adjudicating complex cases cement jurists’ authority and constitute an insurmountable difference to their own status in the courthouse. It is not unusual for them to express feelings of respect and occasionally of awe and deference during interviews. Consider the following excerpt from my interview with Julia, the owner of a bakery in a small city:

What one feels is that one is like a pupil, a disciple, in a certain way in an inferior position in the sense that [judges], obviously, are people who studied so much. They also have a lot of experience in their roles, and for us it is the first time, obviously. We are newcomers, we are nothing or nobody in the sense that; I mean this in relation to their professional position, not as persons. Then, beyond having the freedom to say guilty or innocent, one knows that, ultimately, they are the ones who are in the know.

In one way or another, most of jurors’ narratives transpire a sense of ambiguity (Amietta, 2021). On the one hand, they are *in situ*, in direct contact with the workings of law at one of its very powerhouses, and invited to take part in its gearing mechanisms. On the other hand, even after having saved the spatial and temporal distances that separate their ordinary lives from the law, jurors are made to remain alien to much of its rationale and working principles. Behind these feelings is the law’s very ability to present itself as ahistorical and immutable in the face of the petty actions of individuals. Legal professionals’ skills to turn those individuals’ stories into judgable legal artifacts (Bergman Blix and Minissale, 2022) with a performance of objectivity (Bourdieu, 1987, 2003) mark their separation from the sphere of the law. The distant law works, in this sense, as a convenient counterpart that helps jurors locate themselves and their role in legal proceedings when they recall it in their interviews. Pitting their own trajectories against this understanding and performance of law, jurors readily identify with the ‘common sense’ realm. Natalia, who works in real estate in the Capital City, put it in these terms:

How would I describe being a juror? I think it's the view of the people, the point of view of the people. It’s not one of the theoretical knowledge or the formality of the process but it’s to see how those two points of view can eventually come together. A person who […] has nothing to do with the justice or with the law, a person who never took part of something like this in their life, can end up having the same opinion as a judge […]. It is, for me, the point of view of the people, of common people.

Jurors’ perception of the law as distant and their self-identification with common sense does not mean that they do not make use of proper legal resources. Jurors use legal jargon, and their narratives convey their efforts to make legally informed decisions—embracing the legitimizing claim to objectivity and attachment to legal proceedings that is commonplace for legal professionals. This was best illustrated in their narratives about justice, where the language of the law appeared recurrently and much more so than any reference to substantial fairness more closely associated with a commonsensical understanding of justice (Finkel, 2001). The very jurors, who identified as representing ‘common sense’ in the courts, readily evaluated the outcomes of cases, resorting to conceptions of justice infused with legal jargon and arguments. The emphasis was on procedural correctness, even in cases where they did not agree with the substantive outcomes of the case. Consider the following excerpt from the interview with Julio, an IT specialist and owner of a shop in a small town:

Positively, surely yes [justice was made]. Because I repeat, it doesn't matter if a person is guilty or not. If the evidence is not enough [for a guilty verdict], it means the justice system acted correctly. It isn’t about judging whether the person committed the crime or not. But if all the evidence presented, the testimonies and everything that comes with it, condemn someone, then they must be convicted. And if there are doubts, like in this case there were many doubts, then they must be acquitted.

I followed Julio’s assertion by asking if justice could be said to have been served after the crime had gone unresolved. Julio went on: “Well, that is the thorn I’ll always have in my flesh. Because I have not had news, I did not try to find out either, nor did I research on the Internet, if at some point the culprits were found.” The law can be said to have proved useful in providing jurors with a sense of justice and the idioms to narrate their own (often dispassionate on this point) stories of making justice, even if incomplete and burdened with the limitations of the law’s content and proceedings. This sense of justice works to set the boundaries between a legitimate force, that of the law, and the force that is mere violence, the force of the criminal (Derrida, 1992, pp. 6–7), see also Valverde 1999. When overtly spoken about by jurors, justice is largely a retributive force that reflects the ideals of modern liberal law in terms of proportional deserved punishment. Importantly, jurors do not only *legitimate* the law’s own violence through their very participation in the decision (Sarat, 1995). They also enthusiastically deploy the legal version of justice as proportional, legally defined, objectively assessed and impartially inflicted retributions as they go about telling their stories and making sense of them. The law is distant and inaccessible, but it provides tools to deal with matters of subjectivity making, relationality and emotions, as a prop to conveniently locate power and violence in that authoritative but remote source—and it does so equally for legal professionals and laypersons.

## 3 Jurors making legal sense: with, against or beyond the law?

For most of the jurors I interviewed, the law remained a remote, if respectable, source of authority—authority that can, in turn, be readily used to shield oneself from difficult questions on a difficult decision. Juror’s participation, even if marked by a commitment to attentive involvement, is rarely perceived as one that left an imprint on, engaged with, or resisted against the previously patterned paths of legal workings. But jurors also told stories of engaging in the creative games of the law, and of consciously using, subverting or going beyond legal mandates to pursue their agendas in ways that problematize the law/common sense, lay/expert and emotion/reason dichotomies and complicate the task of empirically discerning the contours of judicial objectivity in practice.

Let me begin illustrating this point with the case of Norma, a businesswoman who took part in the divided decision of a murder trial. The case considered a police officer who killed a teenager with a supposedly non-lethal gun. The victim was from a very low-income family living in an area of the Capital City that faced multiple challenges and deprivation. He had a history of conflict with the criminal law linked to substance use disorders and had been institutionalized in the past - mostly in relation to petty theft and assaults. His family had called the police as he was on the roof of the house, wielding a knife and shouting threats. After the police asked the boy to come down, he jumped from the roof toward one of the officers, who shot him in the chest with a rubber bullet, ultimately causing his death. Consider the narration of Norma:

At that moment, after listening to the witnesses from both sides, I wasn’t judging a person. I was judging an institution. Because I put myself in [the police officer's] shoes. He gave this person, who was high on drugs, a warning. He was armed with a barbeque fork and knife. […] I don't know if he just wanted to come down, he was high on drugs, and I don't know about drugs […] I don't know the smell of drug, but drugs make me desperate. This was a young man, he jumped on him; he shot the rubber bullets gun, with such bad luck that the reverberation ripped his heart. Then I put myself in his shoes. I told the judges ‘I’m not judging a person; here I am judging an institution. If I condemn him, what will the police as an institution do when I call them because they are robbing me, or they are raping my daughter? They’ll go around the block ten times, and then they’ll come, once they’re gone, because they’ll be afraid of shooting, they’ll be afraid of proceeding. Then I am judging an institution.’ ‘But Norma, you can’t’, the judge insisted. 'Let’s give him a minimal punishment but give him a punishment because he killed a person’. But I didn’t want that, and I voted that way. They didn’t manage to change mine or two other jurors’ opinion, because I’m judging an institution, I can’t declare him guilty. He didn’t shoot with a fire gun, he shot with what the police told him to shoot, with the rubber bullets gun. Then I said: ‘Innocent’. Do you know what the judges said to me? ‘Norma, this can’t be done, he’s been in prison for two years, what do we do with those two years?’ Look at the influence of that. They gave him the minimum sentence, he’d be released in a month, but they didn’t want to discharge him due to those two years they had him in. I didn’t care about that.

Carlos, a retired history teacher and a member of the mixed tribunal in this trial, took the opposite stance but also did so by stretching the limits of what he considered the proper legal outcome. Complaining about jurors who wanted to “blame the dead one,” he voted guilty. He did, however, recognize that he did so because the family of the victim was poor, and a guilty verdict would secure them “an economic compensation, at least,” the costs of which would be “in charge of the Province, as [the defendant] was a police officer.” He described this as “a Solomonic thing, the man was in prison for that time, and the family gets recognition of the economic part.”

The monolithic image of the law as distant and immune to non-legal influences fades in these jurors’ stories. Closer to what Ewick and Silbey theorized as ‘With the Law’ legal consciousness (Ewick and Silbey, 1998, pp. 131–132), these narratives point toward the deployment of legal and extra-legal resources to engage in knowledge games that imply an understanding of the law itself as flexible and accommodating to political struggles. Norma advanced her own agenda, which went well beyond the case being decided. She resisted against legal professionals’ position with a mix of arguments that blended police officers’ shooting training with her concerns about security and fears of future victimization. Norma’s case could readily be coded as an example of commonsensical justice’s flexibility in remedying the objectively legal outcome (Finkel, 2001, pp. 319–330). But her story is not one of a clash between two forms of making justice. She does resist against an outcome that is unfair due to too inflexible an application of the law. Quite the opposite, she denounces the judges for being in control of law’s *plasticity* and *totality* (Latour, 2010) and, as such, being able to negotiate a more convenient outcome by means of the manipulation of law’s technicalities. For Carlos, in his turn, the decision was not a place where emotions did not belong. The process involved his dislike for reactionary political ideas and his empathy with a grieving poor family in need of relief. From opposing political stances, both acted tactically and, with the resources at hand, tried to make use of their opportunity to curb the direction of a decision they saw could be unfair. The affective components of legal decision-making were at the forefront of their story of ‘making justice’, but neither of them situated the judges’ decisions in the place of the objective ‘legal’ resolution of the case against which they resisted. In fact, they seem to imply that such a resolution probably did not exist.

Ewick and Silbey complete their well-known triptych of the cultural schema of citizen’s relationship with the law with overt contestation and resistance—what they term ‘against the Law’ legal consciousness. Instead of respectful deference or tactical maneuvering, ordinary citizens stand here “up against” the law (1998: 180). Jurors I interviewed experienced situations they deemed unfair or hard to understand, but overt opposition to the law does not capture what they describe as their stance in such instances. They did not intend to “[pass] the message that legality can be opposed” (Ewick and Silbey, 1998, pp. 49). On the contrary, most open contestations in our interviews came in the form of demands for the law to fulfill its promise: to act as an objective, predictable set of rules—even when jurors do not necessarily know what these rules are. I will term this stance *beyond the law*. Let me start illustrating this point with an excerpt from my interview with Camila, a university student and retail worker in her thirties who participated in two cases. Camila insistently described her perplexity with some of the workings of the law during her time at the courthouse:

Camila: Beyond [the outcome of the trial] being logical, I mean, there was no other choice, but there wasn't evidence to say 'yes, it was [the defendants]'. You know what I mean? I mean, they were supposed to be guilty, so, from five defendants, they convicted three. But they didn’t sentence them to many years [in prison], because they did… Look at what [the judges] did: they judged that two of [the defendants] went [to the murder location] already determined [to kill] and the third one was just accompanying. Because otherwise if they ruled that three of them came there with the intent to kill, it would have been… premeditated, is it?

SA: Possibly that you conspired to kill someone?

Camila: And then it would have been life imprisonment, wouldn't it?

The strategic use of legal technicalities by judges here is not dissimilar to the ones attempted by the judges in Norma and Carlos’s story. Camila’s feelings and reactions, however, were different. She did not openly contest the maneuver but dissented, voting for the innocence of one defendant for whose participation in the murder she thought there was no evidence. She felt, and repeated through the interview, that “cases are already half solved” and that the mixed tribunal is just “a sort of formality,” only to “close a case saying, ‘but the people also participated, the jury’“. Her doubts about the law’s mandates and how they do or do not determine the outcome of trials were recurrent throughout our conversation. She had paid thorough attention to details and kept asking me about fine-grained legal rules. The idea of perjury as an offence, for instance, puzzled her:

For example: they say “nobody can lie because otherwise afterwards…” And you see that people lied, that finally the decision was in a way that meant that they didn’t believe in what those people said. But nothing is done about that. Why do they say that? Why do they tell them they can’t lie? The only one who can lie is the defendant, if I’m not mistaken, but the witnesses shall not lie, and they lie and then the decision says that they lied and… What happens there? Nothing. Then why do they do that? It’s like sometimes it’s not clear.

Camila’s mobilization of her (admittedly limited) legal knowledge is telling in that she calls for the law to fulfil its promises of rule attachment and predictability against the very actual workings of the law. The plasticity of the law’s operation is not, in her view, necessarily unfair or biased, and she did not imply a hidden agenda or undue external influences. Yet the ambiguity is puzzling even if it works toward a fairer decision. The central argument of this article is that this problematizes jurors’ depiction as champions of versions of justice linked less to objectivity and procedural impeccability than to substantive outcomes. I will try to illustrate this point further by discussing at some length the story of Ana, who served in a murder trial where the defendant and victim were friends and had been experiencing homelessness. “To me, this created some sort of a contradiction, to take part of something I did not have any kind of competence for,” Ana started the narration of her feelings as she arrived at the courthouse to serve as a juror. She continued:

It seemed to me pretty terrible. I didn’t see why I or any other citizen would have the right to judge someone… it’s as if I had the right to do surgery on a person. I thought it was meddling in a terrain where one has no formation. There are specific courses for one to be able to fulfil specific tasks. In this case, there is an education in law that makes you qualified in law, presumably, and teaches different bodies of knowledge which qualify you to assess evidence and to judge a case. And I didn’t have any formation in that sense.

A philosophy university teacher and doctoral researcher, Ana was an expert in a different field and identified criminal justice as a space that should also be expert-led. This shaped not only her impressions as to the pertinence of lay participation but also the direction of her contestations as she grew increasingly discontented with the way the trial progressed. In line with her ideas, she demanded that judges exercise their pedagogical authority upon jurors:

In my opinion it should have been a task of the judges to make people who are not very much aware of how they should behave, behave as they are supposed to behave. But many of the other jurors said: “well, although I actually don’t know if this is certain or not, it won’t be bad for him to be in prison instead of being in the streets and have the chance to reform and get education; so yes, I consider him guilty”. And then the judge, in my conception, should have functioned as an instructor and said “no, listen to me, our constitution says something else. You have to acquit him, if you don't have conclusive evidence you should vote against, shouldn’t you?” But no, they didn’t say anything. They took note of their vote and it was registered in the decision. […] Everything turned upside down. Their function should have been to explain [the law] to the lay people there who have no formation or knowledge.

The knowledge-ignorance logic of Ana’s argument was not without breaches. Although she put herself on the lay side and repeatedly insisted on not knowing how things were, she argued for what she considered would make the proceedings legally correct. Consider her discussion with judges about deliberation, access to the case file and guilty pleas:

[Prior to the deliberation] you were supposed to see only the two or three pages [of the case file] that were marked by them as relevant evidence, to what I explicitly responded: “you will excuse me but until I don’t read the whole file, I cannot make a decision”. [A judge] got quite annoyed with this and said: “but how can you say that”. “Well, it is my right to see all of this before I can say what my opinion is”. Meanwhile the judge told me: “but he already pleaded guilty”, to which I replied: “In the little leaflet you give us says that if the defendant declares themselves guilty that’s not evidence of anything. So, what do you mean by ‘he pleaded guilty’? I don't care how he pleaded”. So, it was a bit tense.

The argument between Ana and the judges resulted in a delay in the deliberation while she took the next morning to study the file—something, in fact, banned by the law. The exchange of legal and quasi-legal knowledge between Ana and the judges is telling. Ana’s ‘resistant’ stance against legal experts carries the paradox of ‘law’ being brandished by a lay member of the tribunal—even if not with strictly correct arguments—against professional judges. The jurist, in their turn, abandons the default neutralizing distance of the “juridically regulated debate” (Bourdieu, 1987, pp. 812–13) and lets go of their emotions of annoyance. What is most puzzling for Ana and Camila are situations in which the law is perceived as not doing things *legally* enough (Latour, 2010). This is not in the sense of the legal-illegal dichotomy (neither of them is accusing judges of strictly unlawful or biased behavior), but as in the myth of law as an objective, rule-bound, predictable justice-making machine where knowledgeable agents put the proper rules into action and discipline others into doing so as well. Ana’s demand of legal professionals was not to act fairly but to retreat into their particular brand of objectivity so she could herself go back to the status she belonged in. Ana concluded her narration jokingly: “when I arrived, I thought ‘what am I doing here’. By the end of the trial, I was like ‘what are all these others doing here? I should be deciding this on my own’.

Jurors going *beyond* the law, asking for the law to behave more legally than it does, or it can, has been the most common form of contestation among my interviewees and an interesting component of their making (legal) sense of their experience. These *beyond the law* positions do not always take the form of open contestation. Let me return to Julio’s story to illustrate this. He served in a homicide case, and as we saw above in this section, he was certain about the fairness of the defendant’s acquittal. But there was a particularity in the decision of the case. After the hearings finished, the trial prosecutor stated in his final allegations that not enough evidence had been collected and asked for the defendant to be acquitted. The judges informed the jurors that no deliberation or voting was required and that the defendant would be automatically acquitted. Julio was satisfied with the substantive decision and did not have any objections to the procedure. He had perfectly understood the technical intricacies of the situation, and his response to it had been devoid of much emotional load. His reaction to the unexpected denouement, however, entailed the creative use of his own take on legal technologies:

[The deliberation] was quite explicit, quite fast, because when the prosecutor does his allegation and admits that the evidence presented is not enough, practically there was nothing else to say. All of that with the consent of the judges who gave the same opinion. There was no need to issue a verdict as a public jury […] Anyhow, I presented a document where… As I was updating my diary on a daily basis and my opinions were continually going there, I presented it [to the court] because it was done, and I also wanted to prove that I was committed to what I was doing, and I wanted my opinion to be known. I submitted a copy to the judge. The judge made it public to the rest of the jurors. So, we didn't need to issue a verdict, but I gave my opinion anyway.

Julio gave me a copy of his written decision, a four-page document introducing the facts of the case, analyzing the evidence, weighing the credibility and pertinence of the testimonies, and reaching a non-guilty conclusion. It could, in substance and, with minor tweaks, in form, have well passed off as a judicial decision. It had a signature, a printed name and an ID number, and below Julio’s name, it read *Jurado Popular* (lay juror). Argentina’s criminal justice systems’ slow transition to oral procedures has been a matter of much debate (Bergoglio, 2010; Hammergren, 2007). It is well beyond the scope of this article to engage in those discussions, but Julio’s use of this mechanism of inscription deserves some reflection. The use of a durable vehicle, a document that is archivable and certified with his signature, is a testimony to his deference to the law’s forms, his commitment to an objective assessment of the evidence, and his devotion to his function as a juror. Just as the use of legal jargon—which abounded in Julio’s interview and his written verdict—the deployment of a properly legal inscription device was a way of leaving his imprint in the detached times and spaces of the law. In adding an extra layer of formality to the process with the submission of a written verdict, he also went *beyond* the law. He stretched legality past his legal obligations, which had ceased long before, and was happy and moved about this.

## 4 Concluding remarks

This article aimed to empirically discern the place of law, legal knowledge and judicial objectivity in jurors’ experiences of participation. I argued that such an examination tells us something about the complex cognitive and affective process of developing relational legal consciousness—understood as a set of situated, contextual, experiential understandings of and relationships with the law built at least partially in interactions with others (Nielsen, 2024). The analysis showed, first, that jurors readily embrace common parlance in academic and conventional understandings of lay participation and resort to the shorthand *common sense*, as opposed to the overly bureaucratized, expert-controlled, rule-bound ‘law’, to define their role and place in criminal justice proceedings. As such, they tend to describe themselves as remaining external to that reified, distant world of legality, in what Ewick and Silbey, evoking Kafka’s famous parable, have called a “Before the Law” legal consciousness scheme (Ewick and Silbey, 1998, pp. 74–77). Jurors tend to take the legal networks they are exposed to (and particularly, their promises of formality and predictability) very seriously—more seriously than legal professionals. Jurors speak of an engagement with the law germane to what Valverde described, referring to lay witnesses of North American appellate court proceedings as “a more black-letter manner than senior state lawyers and Supreme Court justices” (Valverde, 2005, pp. 421). It is the law’s plasticity in the hands of legal professionals, not its majesty and complexity, that jurors tend to experience as most puzzling.

This does not mean that jurors do not speak about engaging in playful or resistant involvements with the law and with the professionals who are supposed to guard it. Jurors tell stories of openly challenging this authority to pursue alternative agendas, and they do so in terms that can be readily coded as bringing ‘common sense’ to the—overtly political in these cases—decision of criminal cases. However, in instances in which conflict is talked about, it was more common to hear them speaking about their deployment of legal and quasi-legal knowledge and insisting that the law more thoroughly performs its brand of objectivity. Open contestations mostly took the form of calls to reinforce the law’s neutrality and formalism—to rescue it, not necessarily from illegality, but from the law’s own elasticity. As such, I have argued that jurors’ stories of the use of legal knowledge and technologies occasionally show them going *beyond* the very obligations (and possibilities) of the law. Jurors told me many things they do with the law—even breaking it—but often with the aim of sustaining the value of a sense of justice bound by legality.

A closing note needs to be made about a point that was not covered in detail in the article. It has to do with the protagonists of the stories I told, particularly the ones of the most active, engaged, and resistant involvements with law and legal professionals. As it emerges from the cases that illustrate my arguments, the chances of telling stories of subversion, creativity, or simply advancing challenges to legal professionals’ stances appear to be related to socio-economic and educational background. In Bourdieusian terms, it is the relationally defined forms of capital attached to these traits that create the conditions that make such interventions possible (Bourdieu, 1977). It is the university graduates, doctoral students, politically engaged educators and businesspeople of my admittedly small sample who mobilized alternative uses of legal and quasi-legal knowledge and reframed judicial objectivity in ways that altered, if not the outcomes of cases, at least the decision-making process. This crucially draws attention to the need to remain vigilant in the discrete contexts of our empirical explorations to the uneven opportunities to deploy both affective and cognitive tools for political action. The point is especially relevant when discussing a very powerful legitimizing idea as *participation*.

## Data Availability

The datasets presented in this article are not readily available because of ethical reasons. The author is happy to be contacted by any researchers interested in other materials, such as interview or observation protocols. Requests to access the datasets should be directed to SA, s.amietta@keele.ac.uk.

## Footnotes

^1^In this article I use the terms ‘lay juror’, ‘juror’, and ‘lay person’ to refer to the members of Córdoba’s mixed tribunal without legal education who are summoned for a single case. Reasons to choose the term ‘juror’ over other options (notably ‘lay judges’) are several. ‘Juror’ is the closest translation for jurado, which is the expression used in provincial legislation as well as in media, emerging common parlance and academic publications on Córdoba’s 2004 mixed tribunal (jurado popular is indeed the most common expression). The discussion dates back to 1987, when the Province’s constitution was first reformed to allow for lay participation, and the spokesperson for the proposing party said explicitly the term would make it easier to understand and accept, due to familiarity through US-influenced popular culture. Other authors have also used the expression in English for systems of lay participation other than the traditional 12-member juries (see, for example, Hans and Germain, 2011, on the French Cour d’assises, one of the inspirations of Córdoba’s tribunal). ‘Lay judges’ seems to be more common in systems where citizens without legal education are appointed for an extended period of time – like German Schöffen and similar systems in Sweden or Denmark -; Córdoba shares the Civil Law tradition of these Continental systems, but its lay decision-makers are appointed for a single case, much like in classic jury systems.

^2^The research project and procedures for the selection and recruitment of participants and observations at courthouses were approved by the University of Manchester Research Ethics Committee and were conducted in accordance with the agreed ethical protocol. For the recruitment of jurors, I used a combination of purposive, random and snowballing sampling. I relied on lists of jurors provided by the Jurors’ Office of the Judiciary (JO) to contact specific individuals (for example, those who had been part of dissenting decisions). This was complemented with randomly chosen individuals, selected by their order in the JO lists via online random list generators. I relied on telephone calls to initiate contact. I introduced myself to the listed person and immediately made clear how and why I had got their numbers. I proceeded to explain my work and the reason for my call. If the person refused to participate in an interview, I apologised and guaranteed they would not be contacted again, and their data would not be used for any other ends. If the person expressed willingness to participate, I asked for an email or postal address to send a formal invitation and information letter. I suggested any potential participant to read the documents carefully, take time to think about their participation and contact me to ask any questions or arrange the interview if they were still willing to do so, in no less than two weeks’ time. This was considered an ethically satisfactory procedure considering that my first contact would occur through telephone. In locations smaller than the capital city, court officials often accepted to hand copies of my invitation and information letters to jurors at the end of cases to contact me afterwards if they were interested in being interviewed. Snowballing was also used for juror recruitment: I contacted one juror through common acquaintances, and three others through referrals from judges or court officials (again, mostly in small towns). Finally, also in a small city, a juror who had accepted to be interviewed asked whether her friend who had also served as a juror could join the interview. After making sure both were properly informed and freely giving consent, I interviewed those two jurors together. This was the only group interview I conducted with jurors. I conducted all interviews personally and obtained informed consent from all participants. All juror interviews took place during the first period of fieldwork. Interviews lasted between 50 min and 2 h and 45 min, and all of them were completed in a single encounter. Most of them took place in public spaces of mutual convenience, like cafes. Some were done in an office facilitated by a research center from the National University of Córdoba, and in the houses or workplaces of interviewees. Although criminal trial hearings are public in Córdoba, I sought permission to observe hearings from presiding judges, informed parties of the nature of my role and the reason for my presence, and handed summary information letters to individuals involved whenever possible, including jurors, clerks, and police officers. I also informed parties that I would not conduct any observation if they objected to it, although this was never the case. Permission to conduct observation in public and certain private spaces of courthouses was, as is often the case in institutional ethnographic research, a dynamic and ongoing process. Informal conversations and other interactions in the courthouse were used for this research only after consent from the participants was obtained *via* summary information letters.
